# Case Report: Uterine mesothelial cyst: a report of three cases and literature review

**DOI:** 10.3389/fmed.2026.1800692

**Published:** 2026-06-23

**Authors:** Jianfeng Huang, Wenxuan Yang, Yuqing Wang, Weiqing Huang, Daiyu Zhu

**Affiliations:** Hubei Provincial Key Laboratory of Occurrence and Intervention of Rheumatic Diseases, Hubei Provincial Clinical Medical Research Center for Nephropathy, Minda Hospital of Hubei Minzu University, Hubei Minzu University, Enshi, Hubei, China

**Keywords:** diagnosis, postoperative pathological result, therapy, ultrasonography, uterine mesothelial cyst

## Abstract

Mesothelioma cysts are benign tumor-like lesions derived from mesothelial cells. They mainly affect the peritoneum of the pelvic and abdominal cavities, and are more commonly found in the mesentery and greater omentum, while they are less likely to occur in the uterus. The following is a report on the clinical data of 3 rare uterine mesothelial cysts that were treated at Minda Hospital of Hubei Minzu University over the past 10 years. By reviewing and analyzing the treatment processes of three patients and conducting a literature review, this study aims to explore the diagnosis and treatment methods of uterine mesothelial cysts, with the expectation of enhancing gynecologists’ understanding of this disease and avoiding misdiagnosis and missed diagnoses.

## 1 Introduction

Mesothelial cysts constitute a form of cystic mesothelioma. They are considered benign, encapsulated lesions rather than malignant tumors. Typically small and solitary, these cysts are also referred to as isolated mesothelial cysts. In women, uterine mesothelial cysts are relatively uncommon, present with atypical clinical features, and are frequently missed or misdiagnosed. The precise incidence remains unclear, and no unified diagnostic or therapeutic standard exists. This report describes three cases of intrauterine cysts managed via laparoscopic exploration, all with favorable postoperative outcomes.

## 2 Case presentation

### 2.1 Example 1

**Figure 1:**
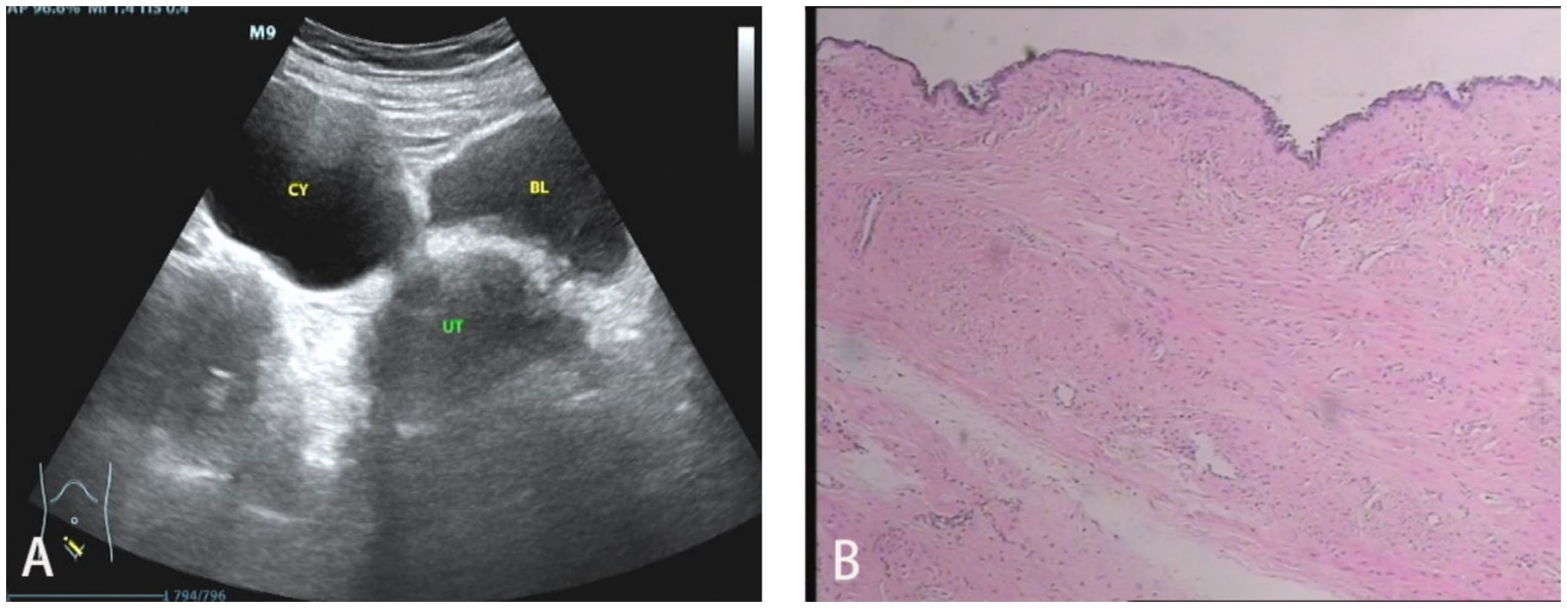
**(A)** Ultrasonography image (UT: Uterus; BL: Bladder; CY: Cyst) and **(B)** postoperative pathological result of the first patient.

The patient is a 42-year-old woman admitted for evaluation of a pelvic mass discovered on routine examination 1 month prior. A pelvic ultrasound at that time revealed a 75 mm*60 mm anechoic cystic mass anterior and to the right of the uterus (Figure 1A), though she reported no abdominal pain or distension. The patient had kidney stones for over 10 years. Gynecological examination indicated the uterus was normal in size, and a smooth, mobile, non-tender mass approximately 6 cm in diameter with well-defined borders was palpated in the right adnexa; the left adnexa was unremarkable. Tumor markers, blood routine, liver and kidney function were normal. Laparoscopic exploration demonstrated an abnormal uterine contour and identified a 7.0 cm diameter cyst on the right fundus with an intact, smooth capsule and no adhesions. The cyst was excised in its entirety, revealing a smooth inner wall and pale yellow clear fluid without papillary nodules. The final pathological diagnosis was a uterine mesothelial cyst, with focal areas of active epithelial cell proliferation (Figure 1B The capsule is covered by a single layer of flat squamous epithelial cells, and the stroma is composed of fibrous connective tissue, accompanied by a small number of chronic inflammatory cells). Postoperative recovery was good and there was no recurrence.

### 2.2 Example 2

**Figure 2:**
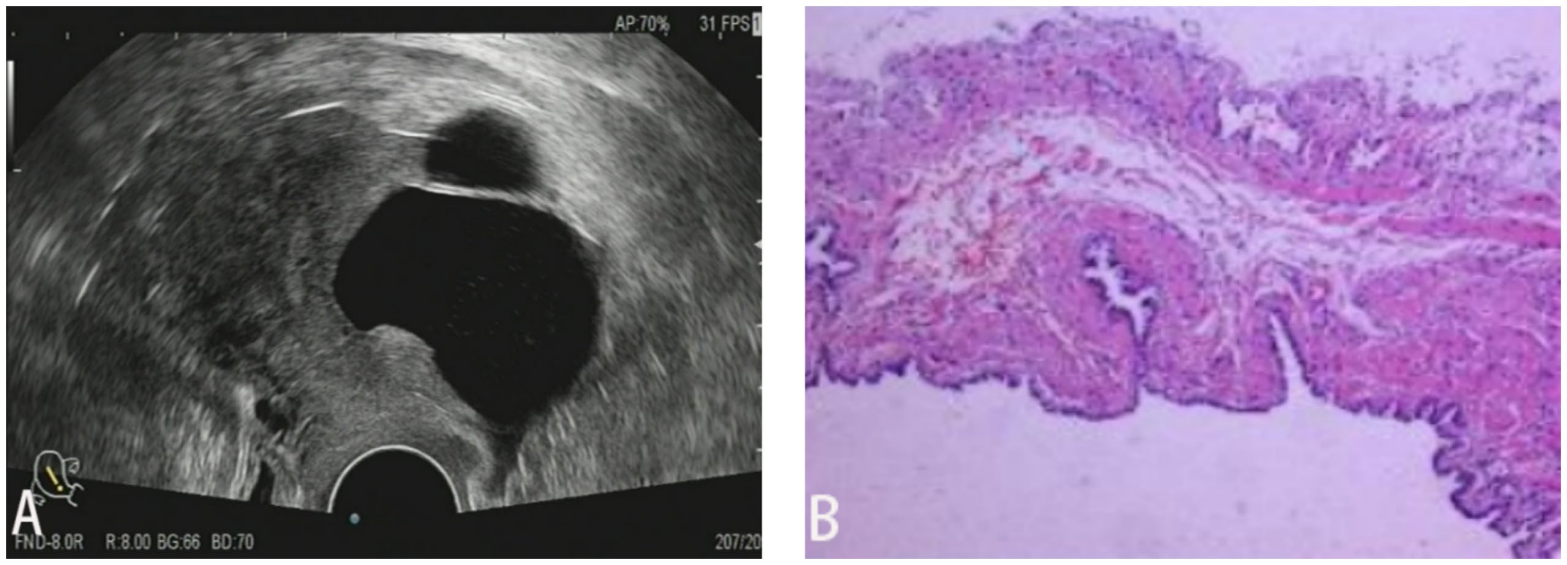
**(A)** Ultrasonography image and **(B)** postoperative pathological result of the second patient.

A 41-year-old female patient presented with a 4-month history of intermittent, dull lower abdominal pain. The pain began without an identifiable cause and was not associated with fever, nausea, vomiting, diarrhea, constipation, or urinary symptoms. Pelvic ultrasonography revealed a 6 mm endometrial thickness and an anechoic area on the posterior uterine wall, measuring approximately 48*32*45 mm, which raised suspicion of a uterine cyst or liquefied malignant tumor due to its poor transparency and peripheral blood flow signals (Figure 2A). The patient had a prior history of one cesarean section. Gynecological examination indicated a normally sized, non-tender uterus, slight thickening of the right adnexa without tenderness, and no palpable masses or tenderness in the left adnexal region. Tumor markers, blood routine, liver and kidney function were normal. Laparoscopic exploration demonstrated an abnormal uterine contour and a smooth, approximately 5 cm diameter cystic mass on the right posterior uterine wall that was not adherent to surrounding tissues; the bilateral fallopian tubes and ovaries appeared normal. The cyst was completely excised and was found to have a smooth wall containing pale yellow, clear fluid. Postoperative pathological examination diagnosed a uterine mesothelial cyst (Figure 2B, The capsule is covered by a single layer of columnar epithelium, accompanied by papillary folds, and the stroma is a fibrous vascular tissue). Postoperative recovery was good and there was no recurrence.

### 2.3 Example 3

**Figure 3:**
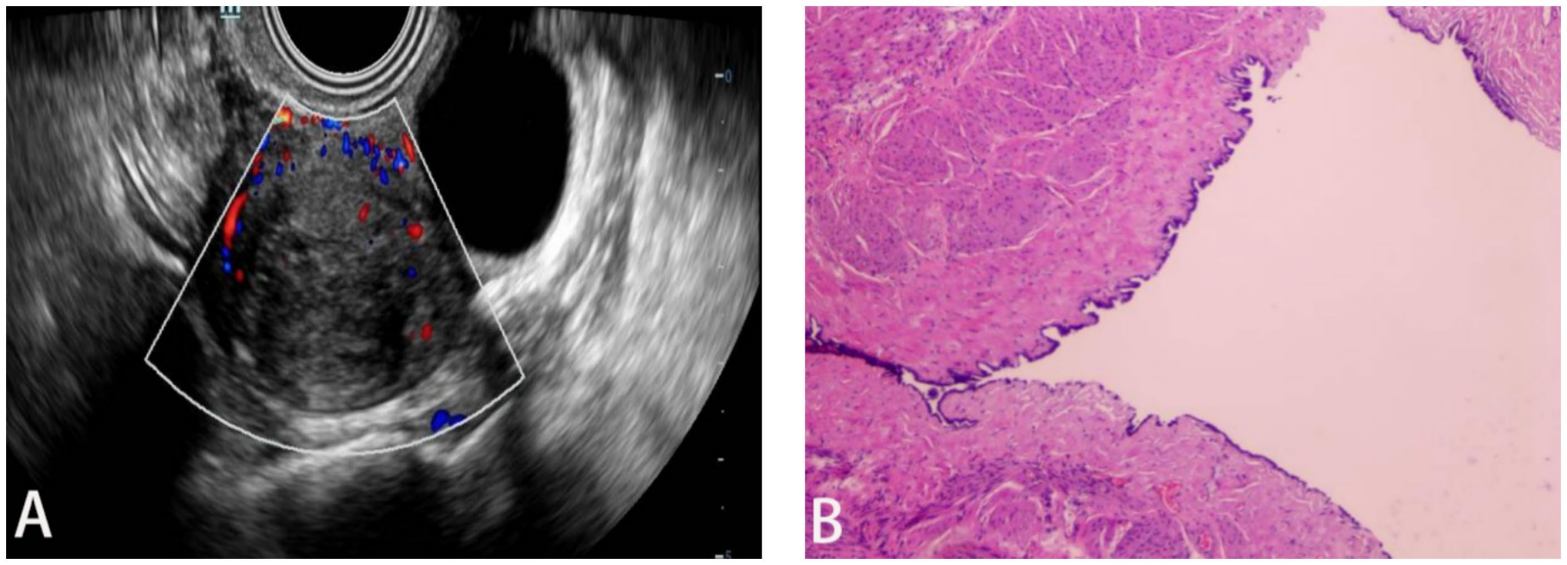
**(A)** Ultrasonography image and **(B)** postoperative pathological result of the third patient.

A 56-year-old female patient was admitted following the incidental discovery of a pelvic mass during a routine medical examination 3 months prior. No intervention was undertaken at that time. Subsequent pelvic ultrasonography revealed a perimenopausal uterus and a multilocular cystic mass on its left side, the nature of which was indeterminate and considered a possible tubal hydrocele (Figure 3A). The patient had a prior history of uterine fibroid removal with unilateral salpingectomy. Gynecological examination indicated slight uterine enlargement and thickened left adnexa without tenderness, while the right adnexa were unremarkable. Tumor markers,blood routine, liver and kidney function were normal. Laparoscopic exploration demonstrated a uterus equivalent in size to a 2-month gestation, featuring a 2-cm diameter, smooth-surfaced fibroid on the anterior wall and a 6-cm multilocular cyst on the posterior wall; the left fallopian tube was absent. The posterior wall cyst and the anterior wall fibroid were completely excised. Postoperative pathology confirmed a periuterine serous cyst and a uterine smooth muscle tumor with hyaline degeneration (Figure 3B, The capsule is covered by transitional epithelium, and the stroma consists of smooth muscle and fibrous vascular tissue, with a small amount of chronic inflammatory cells infiltration). Postoperative recovery was good and there was no recurrence.

## 3 Discussion

Mesothelial cysts can occur on any abdominal membrane surface, including the round ligament, adnexa, mesentery, and peritoneum (1). As a form of cystic mesothelioma, these lesions are benign colloid cysts rather than malignant tumors. Typically solitary and small, they usually measure less than 5 cm and are also described as isolated mesothelial cysts. The cysts are often unilocular, though they may contain two or three cystic cavities. Their walls are very thin and lined by well-differentiated flat mesothelial cells without significant mesothelial hyperplasia (2). This condition is relatively uncommon in women. We identified nine cases of uterine mesothelial cysts after Pubmed search and China National Knowledge Infrastructure (CNKI) published nearly 20 years (Tables 1, 2). Its exact incidence remains unclear, and no unified standard exists for diagnosis and treatment.

**Table 1:** Case reports.

Serial number	Age (years)	Medical history	Presenting complaint	Imaging examinations	Intraoperative findings	Postoperative pathology
1	42	kidney stones for over 10 years	a pelvic mass discovered on routine examination 1 month prior	a 75 mm*60 mm anechoic cystic mass anterior and to the right of the uterus	an abnormal uterine contour and identified a 7.0 cm diameter cyst on the right fundus with an intact, smooth capsule and no adhesions. The cyst was excised in its entirety, revealing a smooth inner wall and pale yellow clear fluid without papillary nodules.	a uterine mesothelial cyst, with focal areas of active epithelial cell proliferation
2	41	a prior history of one cesarean section	a 4-month history of intermittent, dull lower abdominal pain	a 6 mmendometrial thickness and an anechoic area on the posterior uterine wall, measuring approximately 48*32*45 mm, which raised suspicion of a uterine cyst or liquefied malignant tumor due to its poor transparency and peripheral blood flow signals.	an abnormal uterine contour and a smooth, approximately 5 cm diameter cystic mass on the right posterior uterine wall that was not adherent to surrounding tissues; the bilateral fallopian tubes and ovaries appeared normal. The cyst was completely excised and was found to have a smooth wall containing pale yellow, clear fluid	a uterine mesothelial cyst.
3	56	uterine fibroid removal with unilateral salpingectomy	the incidental discovery of a pelvic mass during a routine medical examination three	a perimenopausal uterus and a multilocular cystic mass on its left side, the nature of which was indeterminate and considered a possible tubal hydrocele.	a uterus equivalent in size to a 2-month gestation, featuring a 2-cm diameter, smooth-surfaced fibroid on the anterior wall and a 6-cm multilocular cyst on the posterior wall; the left fallopian tube was absent. The posterior wall cyst and the anterior wall fibroid were completely excised.	a periuterine serous cyst and a uterine smooth muscle tumor with hyaline degeneration

**Table 2:** Case series of uterine mesothelial cysts in the literature

Author, year of publication, references	Presenting complaint	Age (years)	Medical history	Imaging	Interventions	Intraoperative findings	Follow-up
Yu et al., 2014 (10)	Discovered a pelvic mass for over 2 months	49	Unknow	The size of the right ovary is 7 cm × 6 cm. Inside, there is an anechoic area measuring 6.5 cm × 4.8 cm with good transparency and no blood flow signal.	Laparoscopic pelvic mass resection surgery	A mass protrusion is observed on the right posterior wall of the uterus, measuring approximately 9 cm × 8 cm. The top is cystic and there is a flow of yellow, clear liquid.	No abnormalities were observed during the 6-month follow-up after the surgery.
Su et al., 2022 (11)	Discovered pelvic cysts 1 and a half years ago	42	A cesarean section	A 11.2 cm × 6.6 cm × 7.1 mm cystic dark area was observed on the right anterior wall of the uterus, protruding outward. Inside, there were partitions and fine, dense speckled echoes, and a small amount of blood flow signal was detected on the partitions.	Laparotomy	The uterus shows irregular enlargement, resembling the state at 3 months of pregnancy. The anterior wall and the fundus of the uterus present as cystic protrusions, with thin cystic walls. No abnormalities are observed in the bilateral adnexa.	No abnormalities were observed during the 2-year follow-up after the surgery.
Ni et al., 2023 (12)	Discovered a pelvic mass	46	Unknow	On the left side of the uterus, a 4.4 cm × 2.3 cm × 4.1 cm separated cystic mass can be observed. Some of the cyst fluid is clear, while some is not very clear. Blood flow signals can be detected on the cyst wall and the septum.	Surgical treatment	On the upper part of the posterior wall of the uterus, multiple cysts were observed. The surfaces were smooth. Three of the cysts had diameters of 2.5 cm, 2 cm, and 3 cm respectively, and their interiors contained pale yellow and clear fluid.	Unknow
Lin et al., 2024 (8)	Experiencing recurrent menstrual cramps over several years	39	A cesarean section	two adjacent cystic lesions (measuring 4.4 cm 3.6 cm and1.7 cm 1.6 cm) ontherightside of the pelvic cavity; the lesions had infiltrated the adjacent poste rior uterine wall, and did not contain solid components inside them	Laparoscopic uterine cystectomy	This multicystic complex was detected on the surface of the posterior uterine wall during surgery, and it was filled with clear serousfluid	no recurrence was observed at the 1-month follow-up
Mishra et al., 2016 (1)	Abdominal pain for 6 years	40	A cystic mass with a compartmentalized structure (10.3*5.4*8.8cm) on the left side of the pelvic cavity	Left sided ectopic hypoplastic kidney and mild splitting of pelvicalyceal system in right kidney. Liver, pancreas and gall bladder were normal. There were suspected endometriotic changes in uterus with peripheral myome trial cysts with pelvic congestion. Endometrium was 11 mm in thickness and echogenic.	Lparoscopic-assisted vaginal hysterectomy	Uterus was normal size, mid-position with a small intramural fibroid of 2*2cm on posterior wall. There were 9–10 tense clear cysts of 2–2.5 cm in size on posterior serosal surface of uterus. There was no such cyst on adnexa or on any other site.	Recovered well and is asymptomatic
Arislan et al., 2024 (16)	Abdominal pain	41	One cesarean section	An enlarged uterus with a hypoechoic intramural cystic mass measuring 7.2*3.3*3.6 cm in the posterior uterine corpus. The endometrium appeared thin and regular, and the bilateral adnexal regions were unremarkable.	Total laparoscopic hysterectomy and bilateral salpingectomy	Uterus revealed a 6 cm cystic lesion containing serous fluid within the posterior uterine wall	No pathology was observed
Ren et al., 2023 (13)	Self-discovery of a mass in the abdomen for 1 week	27	Never had sexual intercourse and had no abdominal pain, mal uterine bleeding, or	A pelvic anomaly consisting of a multiloculated cyst measur ing 8.9 *8.2 cm, and no solid component was seen	Exploratory single-port laparoscopic surgery	A uterine cyst was observed in the posterior wall of the uterine body and contained clear light-yellow fluid. No other abnormalities were observed.	No evidence of uterine cyst recurrence after 2-year
Shao et al., 2025 (14)	A persistent uterine mass	46	One abortion procedure	A hypoechoic mass measuring approximately 90*75 mm in the anterior uterine corpus, characterized by well-defined margins and internal septations, with blood flow within the mass	Surgical treatment	A multilocular cyst filled with clear yellow fluid was discovered in the anterior wall of the uterus, with no other abnormalities detected. The cyst was found near the endometrium and myometrium, exhibiting a smooth wall without nodules.	No evidence of uterine cyst recurrence after 2-year
Mo et al., 2019 (15)	A space-occupying lesion in the uterus	46	Gravida 3, para 1	A cystic mass with a compartmentalized structure (10.3*5.4*8.8cm) on the left side of the pelvic cavity	Laparoscopic exploration surgery	Unknown	3 months after surgery identified a uterine cystic mass of 3.0*2.5*1.7cm.

The pathogenesis of uterine mesothelial cysts remains unclear. A developmental origin is generally suspected, though some researchers attribute the condition to chronic peritoneal inflammation (3). Established risk factors include pelvic endometriosis, a history of pelvic inflammatory disease, and prior pelvic surgery (4). Among the three cases mentioned in the text, two of them had a history of pelvic surgery, which is consistent with the high-risk factor of a previous pelvic surgery. One case had a history of kidney stones for over 10 years, which might be related to long-term abdominal membrane irritation.

Mesothelial cysts lack typical clinical manifestations and are usually diagnosed following the detection of an abdominal mass or discovered incidentally during other abdominal surgeries. Only a minority of patients present with symptoms such as menorrhagia. Both imaging and laboratory findings are non-specific, complicating preoperative diagnosis and frequently leading to misdiagnoses such as cystic degeneration of uterine fibroids, ovarian cysts, inflammatory encapsulated effusions, or pelvic cystic lymphangioma. Cystic degeneration of uterine fibroids typically appears as patchy anechoic areas with thick, irregular walls. Uterine cystic lymphangiomas are often located within the myometrium and may be associated with irregular vaginal bleeding. Endometriosis usually manifests as multiple small anechoic areas containing fine internal echoes, with the surrounding myometrium exhibiting enhanced and heterogeneous echogenicity, frequently accompanied by dysmenorrhea (5). Currently, definitive diagnosis relies on postoperative pathological examination. Microscopically, the cysts are typically lined by a single layer of cuboidal epithelium without hyperplasia, exhibiting a moderate nuclear-to-cytoplasmic ratio, an absence of mitotic figures, and normal cellular polarity. Immunohistochemical markers can aid in distinguishing mesothelial cysts. Calcium-binding proteins demonstrate good specificity and sensitivity for mesothelial cells, while other useful markers include h-caldesmon, cytokeratin 5/6, WT-1, HBME, and mesothelin (6, 7). No single marker, however, provides 100% sensitivity and specificity for mesothelial cysts (8).

Mesothelial cysts are extremely rare in clinical entities, and no clear treatment guidelines currently exist. Given their benign nature, some authors advocate for conservative management with close follow-up when the diagnosis is certain. In practice, however, preoperative differentiation from ovarian tumors, uterine fibroids, and other cystic conditions is often difficult due to a lack of specific laboratory or imaging findings. Consequently, surgical excision remains the primary treatment. Unlike multicystic mesothelioma, which has a high recurrence rate (27–75%) and a potential for malignant transformation, benign mesothelial cysts seldom recur and show no malignant tendency (9). All three cases of uterine mesothelial cyst diagnosed at our institution had a favorable prognosis following surgical resection, with no recurrence observed.

## 4 Conclusion

In conclusion, endometrial seromeningitic cysts are rare and should be included in the differential diagnosis of pelvic lesions. Enhancing the understanding of this rare disease is conducive to achieving individualized management.

## Data Availability

The original contributions presented in the study are included in the article/supplementary material, further inquiries can be directed to the corresponding author.
